# Echography analysis of musculoskeletal, heart and liver alterations associated with endothelial dysfunction in obese rats

**DOI:** 10.1186/s12902-020-00603-7

**Published:** 2020-08-14

**Authors:** Alejandra Martínez Coria, Norma Angélica Estrada-Cruz, María Inés Pérez Ordoñez, Daniel H. Montes-Cortes, Leticia Manuel-Apolinar

**Affiliations:** 1 Hospital de Cardiología, Centro Médico Nacional Siglo XXI, Instituto Mexicano del Seguro Social, Mexico City, Mexico; 2 Unidad de Investigación Médica en Enfermedades Endocrinas, Hospital de Especialidades, Centro Médico Nacional Siglo XXI, Instituto Mexicano del Seguro Social, Av. Cuauhtémoc 330, C.P. 06720 Ciudad de México, Mexico; 3 Departamento de Urgencias Adultos, Hospital General Centro Médico Nacional “La Raza”, Instituto Mexicano del Seguro Social/Coordinación de Enseñanza e Investigación, Hospital Regional 1° de Octubre, Instituto de Seguridad y Servicios Sociales de los Trabajadores del Estado, Mexico City, Mexico

**Keywords:** Obesity, Endothelial dysfunction, ICAM-1, Echographic analysis

## Abstract

**Background:**

Modern imaging plays a central role in the care of obese patients, and there is an integral focus on its use and accessibility in individuals who have alterations of various in various organs. The objective in this study was to perform an echographic analysis of musculoskeletal system disorders, endothelial dysfunction and the left ventricle (LV) in obese rats.

**Methods:**

Sprague Dawley rats (250 ± 5 g) were obtained and divided into two groups: the control (C) group was fed with a standard diet, and the obese (Ob) group was fed hyper caloric diet with a high fructose-fat content for 4 months. Body weight, cholesterol, triglycerides, glucose, inflammatory cytokines and adhesion molecules (ICAM-1, VCAM-1) were measured. Additionally, two-dimensional echocardiography, abdominal ultrasound and musculoskeletal system studies were performed in the lower extremities.

**Results:**

The body weight in the Ob group was increased compared to that in the control group, (*p* < 0.001); in addition, increased glucose, cholesterol and triglyceride concentrations (*p* < 0.05) as well as increased levels of the adhesion molecules ICAM-1 and, VCAM-1 (*p* < 0.01) were found in the Ob group vs the C group. On ultrasound, 75% of the Ob group presented fatty liver and distal joint abnormalities.

**Conclusion:**

Obese rats exhibit endothelial dysfunction and musculoskeletal changes, also, fatty liver and articular cysts in the posterior region of the distal lower- extremity joints.

## Background

Overweight and obesity are defined as an abnormal or excessive accumulation of fat that is harmful to health, and are an important risk factor for non-communicable diseases, such as cardiovascular diseases (mainly heart disease and strokes); moreover, overweight/obesity is associated with metabolic disorders (glucose intolerance, insulin resistance, hyperlipidemia, diabetes and hypertension), musculoskeletal disorders (especially osteoarthritis) and some cancers (endometrial, breast, ovarian, prostate, liver, gallbladder, kidney and colon) [1].

Some mechanisms that are triggered by obesity involve the inflammatory process, which includes the production of proinflammatory cytokines such as interleukin-6 (IL-6) and tumor necrosis factor-α (TNF-α). Thus, the transendothelial migration of inflammatory cells (leucocytes) and the production of cytokines are an early step in endothelial dysfunction, which continues with the activation of adhesion molecules (ICAM-1, VCAM-1); this contributes to the progression from early endothelial dysfunction to atherosclerotic plaques, causing vascular complications [2–6]. To understand these pathophysiological mechanisms, experimental models are used, and these approaches include murine models (rats). Which is valuable tool for uncovering the mechanisms linked to the comorbidities of the metabolic disease [7].

In obese rats, excess lipid accumulates in other tissues, including the liver, skeletal muscle and heart, and this condition is associated with an increase in adipose mass and free fatty acids. Likewise, in the liver, this accumulation along with other intrahepatic signals leads to a derangement in glucostatic and lipidostatic functions, and generate a greater vulnerability to hepatic steatosis [8, 9].

Echography is commonly used in the clinical setting for the diagnosis and follow-up of patients with nonalcoholic fatty liver disease (NAFLD); in addition, this analysis is a good method that allows the examination of arteries, cardiopathies and fatty tissue [10]. However, no study has used murine models to assess the sonographic findings of several organs in obesity, which could be identified as the integration and relationship of dysfunctions in various mechanisms in other organs or comorbidities with in the same organism.

Ultrasound is an economical, accessible, fast, precise, simple, comfortable, and noninvasive procedure that does not cause pain or involve, radiation. In addition, it obtains images with high sensitivity and accuracy and is essential in the study of a variety of organs, such as the liver and the musculoskeletal system. Two-dimensional echocardiography is the study of the heart in two dimensions; it allows us to analyze the organ as a whole and the relationships that the cardiac structures maintain with each other. Two-dimensional echocardiography is very useful for the study of congenital anomalies, the differentiation between thrombi and intracardiac masses, and the analysis of regions that are difficult to access via the one-dimensional echocardiogram [11].

Although there are ultrasound studies in rats where the various morphological and functional aspects are analyzed at the cardiac level, mainly in the left ventricle (LV), none of these studies has been considered important for a joint analysis of both, the LV and the hepatic, renal and musculoskeletal systems represented in humans [12]. Therefore, we herein report the echographic analysis of the left ventricle, hepatic, musculoskeletal disorders and endothelial dysfunction in obesity.

## Methods

### Animals and model of obesity

This was an experimental, cross-sectional and analytical study. The murine model that was used consisted of a population of Sprague Dawley rats strain that were obtained from an inbred colony in the bioterium of the Specialty Hospital of the National Medical Center, Mexican Social Security Institute (Mexico City, Mexico). A total of 20 males weighing 200–250 g, were used.

Animals were randomly allocated and divided into two groups: the control (C) group (*n* = 12) was fed with the standard diet (Formulab 5008 Diet; PMI Nutrition International, Brentwood, MO, USA), and the obese (Ob) group (*n* = 8) was fed with a high fat-fructose diet (standard diet supplemented with 10% lard and 30% fructose mixed and dissolved in drinking water) during 16 weeks’ ad libitum; both groups received this diet until reaching six months of age. Food and water intake, as well as, body weight, were recorded daily during this period. We have worked with this experimental model in other projects [5]. In this murine model the ingestion of a high fat-fructose diet is clearly associated with the development of insulin resistance, disturbed glucose homeostasis and endothelial dysfunction [4–6]. In this work, the obese group had a hypercaloric diet for 4 months, and both groups, C and Ob, were studied at 6 months of age. However, to facilitate other tissue and organ analyses, the feeding continued in both groups until the animals reached 12 months of age, at this time point, the rats were sacrificed via deep anesthesia and the single dose administration of pentobarbital (25 mg/kg, i.p.) [13] (NOM-062-ZOO-1999, revised 2001).

All cages contained wood shavings, bedding and a cardboard tube for environmental enrichment. All rats of each group had ad libitum access to their pellet diet and drinking water and were housed in a hygienically controlled room in groups of 4 rats, in conventional cages at room temperature (22–25 °C), under a light cycle of 12 h’ light/12 h’ dark.

### Ethical statement

All experiments were performed in accordance with the relevant guidelines and regulations of the bioterium of the Specialty Hospital of National Center Medical, of the Mexican Social Security Institute (CMN SXXI-IMSS), in accordance with the Official Mexican Standard (NOM-062-ZOO-1999, revised 2001) for the care and use of laboratory animals. Additionally, we obtained written informed consent to use the animals in our study. This study was approved by the Ethical Committee and the Local Research and Health Committee of the Mexican Social Security Institute (registration number 3601–2015-95). The animals were treated according to the Official Mexican Standard for the care, use and sacrifice of laboratory animals. (NOM-062-ZOO-1999, revised 2001). In this work, the obese group had a hypercaloric diet for 4 months, and both groups, C and Ob, were studied at 6 months of age. However, to facilitate other tissue and organ analyses, the feeding continued in both groups until the animals reached 12 months of age.

### Metabolic parameters and body weight

#### Body weight

The weight in both groups, (group C (*n* = 12) and Ob (*n* = 8)), was recorded from the beginning to the end of the study. Food and water intake were recorded each week.

**Blood samples** were taken at 8 am (during 7 h fasting at 6 months of age) by a longitudinal cut on the end of the tail to measure biochemical blood parameters and inflammatory cytokines. The blood samples were transferred into anticoagulant containing tubes for the measurement of biochemical blood parameters and inflammatory cytokines, respectively. The samples were centrifuged at 5200 g for 15 min. Plasma was separated and stored at − 70 °C until use for bioassay analyses.

Blood glucose levels were determined by placing one drop of blood on blood glucose test strips of the FreeStyle Optium Xceed glucometer (Abbot Diabetes Care Ltd. OYL, UK). Each animal was twice analyzed.

Determinations of the levels of total cholesterol, Triglycerides (TG), high-density lipoprotein (HDL) and low-density lipoprotein (LDL) were made on a Cardiocheck apparatus after placing one drop of blood (see the previous section on blood samples) on a reactive strip, according to the manufacturer’s instructions.

Quantification of insulin and index HOMA-IR, insulin was measured by chemiluminiscence, using an insulin IMMULITE kit (LKIN1 insulin IMMULITE).

Insulin resistance was calculated using the HOMA-IR index with the following formula for the homeostasis model assessment-insulin resistance (HOMA-IR) index: insulin (μU/mL) × glucose (mg/dL)/405.

TNF-α, IL-6, and the adhesion molecules ICAM-1 and VCAM-1 were analyzed using enzyme immunoassay kits with ELISA R&D kit (R&D Systems, Inc., Minneapolis, MN, USA).

### Ultrasound analysis

In this study, the rat was placed in the left lateral decubitus position, with a slight inclination of the head and the transducer was placed in the different acoustic windows. The equipment used was a Philips Affiniti 70 device with a linear transducer of 9 mHz, where the results were obtained in millimeters. During the echocardiographic examination, two cuts were used to evaluate the heart: the left longitudinal parasternal section and the right longitudinal parasternal section. In the left longitudinal sternal section, it is possible to visualize the right ventricle, aorta with aortic valve, left atrium, mitral valve and LV [11].

With rotation of the transducer the right longitudinal sternal section that appears on the left side and the base (atria) that appears on the right side reveal the left ventricular outlet, aortic valve, aortic root and proximal ascending aorta [11, 12].

The measurement of the LV in diastole was performed from the left septal endocardium to the posterior wall endocardium, measuring below the level of the mitral valve. The interventricular septum is located between the mitral ring in its posterior part and the endocardial surface of the high septum in its anterior part. The diastolic thickness of the free wall of the LV was measured, and its value was approximately equal to the diameter of the interventricular (IV).

### Statistical analysis

The data are presented as the means ± standard deviation (SD) of each group. The study groups were statistically analyzed for the difference in means between group C and group Ob with Student’s t test, and a value of *p* < 0.05 was considered to indicate statistical significance. For each study variable (glucose, insulin, lipid profile, HOMA-IR index, proinflammatory cytokines, TNF-α, IL-6, adhesion molecules ICAM-1 and VCAM-1), a statistical analysis was carried out using GraphPad Prism (GraphPad Prism 8 for Windows, San Diego, CA); *p* < 0.05 was considered significant.

## Results

**Fig. 1:**
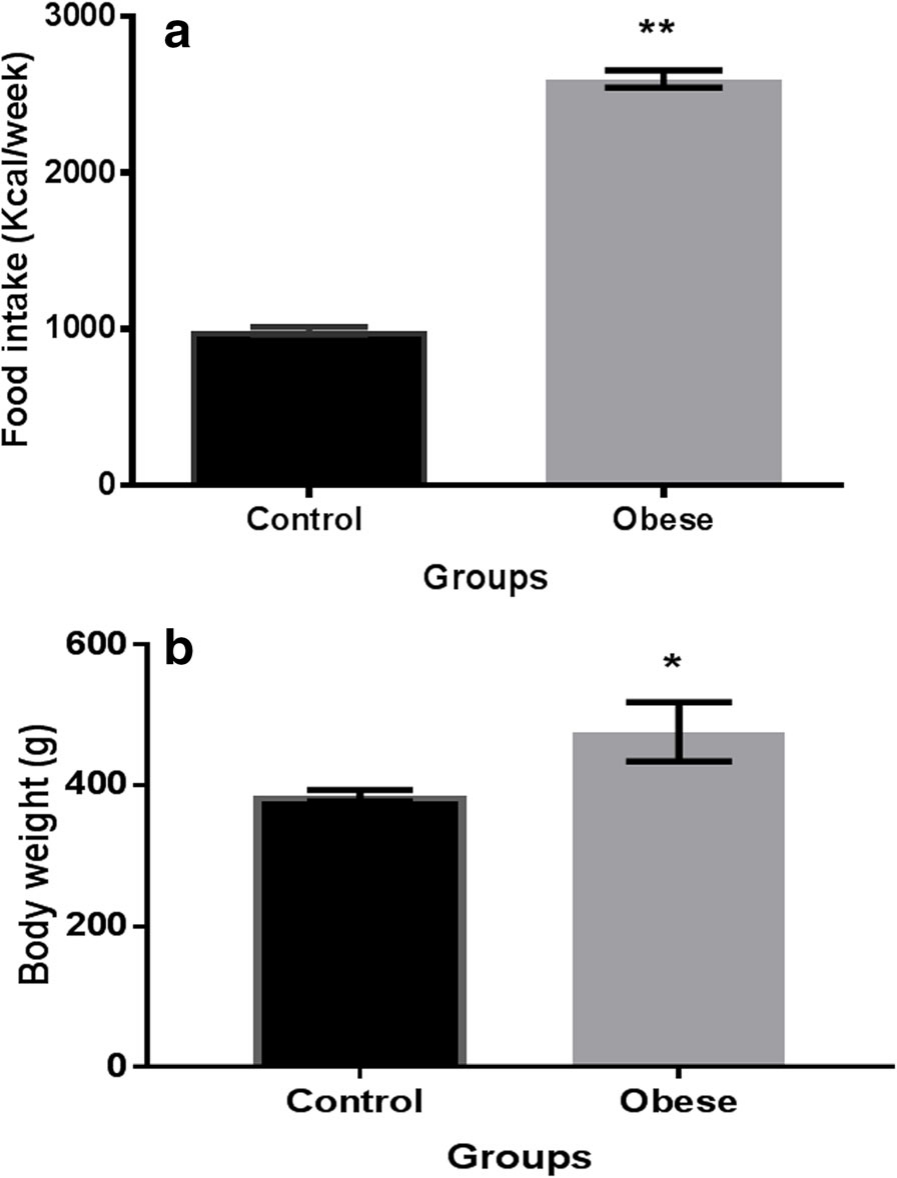
**a** Food intake on the study groups. **b** Body weight in the different groups. Results are presented as values of the mean ± SD, *n* = 8–12 animals per group. Values *p < 0.05, **p < 0.001, Student’s t test, significantly different from the control group

Figure 1 shows the differences in food consumption in the study groups: the obese group fed a hypercaloric diet exhibited an increased food intake compared with that of the control group (*p* < 0.001). Additionally, an increase in body weight was observed in the Ob group vs the C group (22.5%; 472 ± 25 vs 385 ± 20, p < 0.001) and the Ob group showed an increase in fasting glucose level (*p* < 0.05), lipid profile and HOMA-IR index (300%; 4 ± 1 vs 16 ± 3, *p* < 0.0001). Therefore, because of the lipid analysis, these rats were considered obese rats with metabolic alterations (Table 1).

**Table 1:** Metabolic parameters of the control (C) and hypercaloric diet (Obese, Ob) rats

	C *n* = 12	Ob *n* = 8	% difference between Ob and C
Body weight (g)	385 ± 20	472 ± 25**	22.5
Fasting glucose (mg/dL)	69 ± 2	165 ± 45^*^	139.1
HOMA-IR	4 ± 1	16 ± 3^***^	300
Triglycerides (mg/dL)	109 ± 7	225 ± 19^***^	106.4
Cholesterol (mg/dL)	70 ± 5	98 ± 3^***^	40
HDL (mg/dL)	50 ± 5	32 ± 3^***^	−64
LDL (mg/dL)	25 ± 5	58 ± 4^***^	132
VLDL (mg/dL)	33 ± 2	48 ± 4^***^	45.5

Determination of body weight (g), fasting glucose, cholesterol and triglycerides levels in control (C) and obese (Ob) rats. Values are represented as the mean ± SD of 8–12 animals per group. Values of *p < 0.05, **p < 0.001 and ***p < 0.0001 indicate a significant difference from the C group. The % difference is based on a control value of 100%

**Fig. 2:**
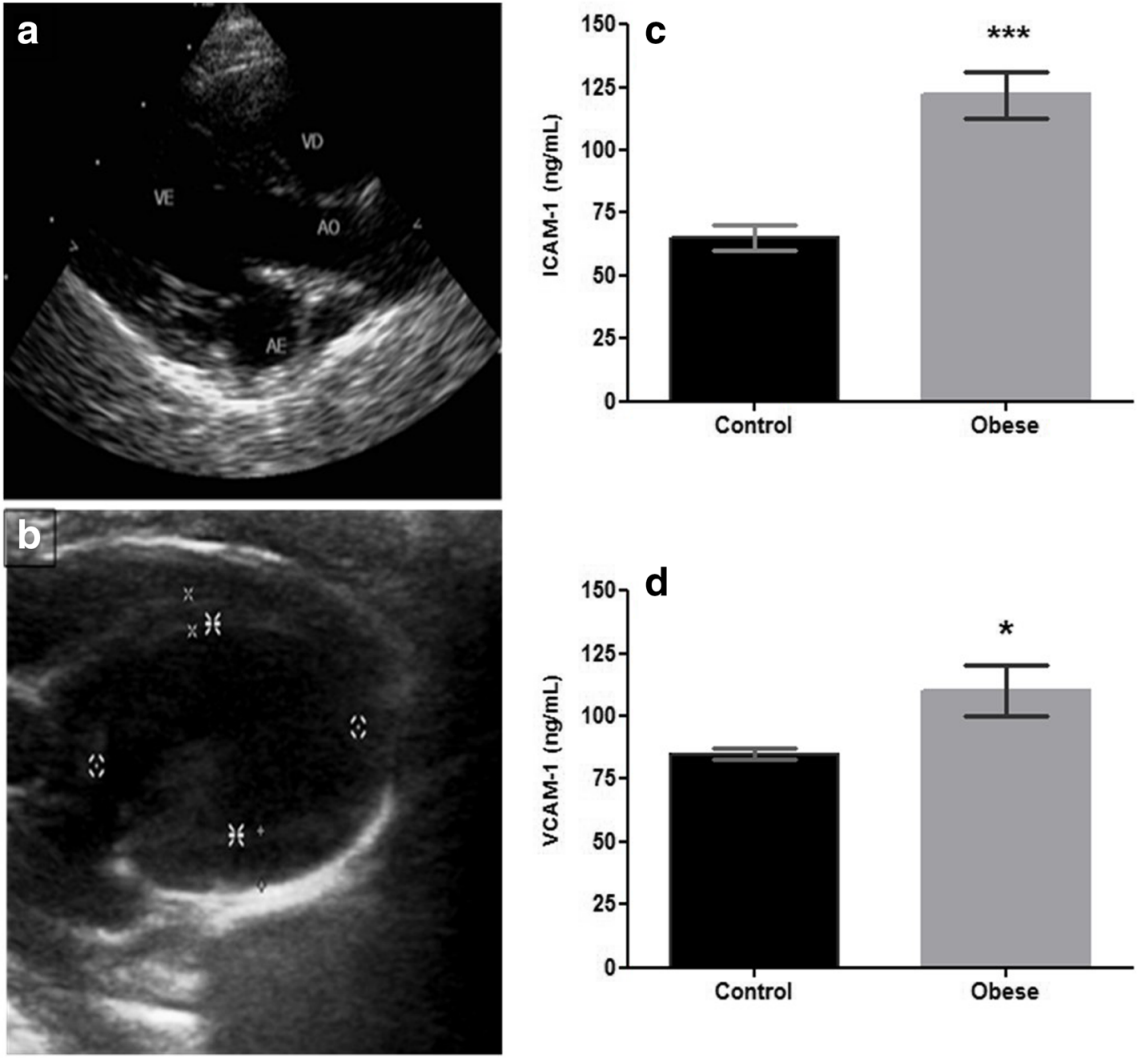
Two-dimensional echocardiography. **a** The left longitudinal parasternal section, where it is possible to evaluate the aorta (AO), the left atrium (AE), and the right ventricle (RV) and left ventricles (LV) in the control group. **b** The approach used for the LV. Markers of endothelial dysfunction such as cellular adhesion molecules (CAMs) in **c** ICAM-1 and **d** VCAM-1 are presented results as values of the mean ± SD

This experimental model demonstrated endothelial dysfunction, manifested by the inflammatory cytokine TNF-α as well as by adhesion molecules that are indicators of endothelial damage, such as ICAM-1 (87.6%, 122 ± 9 vs 65 ± 5, *p* < 0.0001) and VCAM-1 (29.4% 110 ± 10 vs 85 ± 2, *p* < 0.05) (Table 2, Fig. 2c, d). ICAM-1 and VCAM-1 are relevant to chronic inflammatory processes, with an increased risk for type 2 diabetes (T2DM) and cardiovascular diseases (CVD) [6, 7, 14].

**Table 2:** Effects on cytokines and CAMs in groups

	Control *n* = 12	Obese *n* = 8	% difference between Ob and C
TNF-α (pg/mL)	5 ± 0.5	16 ± 2^**^	220
IL-6 (pg/mL)	9 ± 1	23 ± 5**	155.5
IL-10 (pg/mL)	3 ± 1	4 ± 2	33.3
ICAM-1 (ng/mL)	65 ± 5	122 ± 9^***^	87.6
VCAM-1 (ng/mL)	85 ± 2	110 ± 10^*^	29.4

Results are presented as the mean ± SD, n = 8–12 animals per group. Values of *p < 0.05, **p < 0.001 and ***p < 0.0001 indicate a significant difference between the control group and the obese group. The % difference was considered value of control as 100%

In the Ob group, an increase in these adhesion molecules and in proinflammatory cytokines such as TNF-α was demonstrated, with a significant difference compared with the C group (220%, 16 ± 2 vs 5 ± 0.05, *p* < 0.001); a similar increase was observed for IL-6 (155%, 23 ± 5 vs 9 ± 1, p < 0.001) (Table 2). According to the measurements of the metabolic parameters and cytokines, the Ob group, which consumed hypercaloric diet, presented endothelial damage. In addition, modern imaging provided an integral approach to evaluate the control group (Table 3; Fig. 2a, b), in which the aorta and LV were observed. For LV, an increase in the septum and wall was found in the Ob group (Table 3).

**Table 3:** Descriptions of the two-dimensional echocardiographic studies of the kidney, skeletal muscle (lower extremities) and left ventricle (LV), interventricular (IV) partition and LV wall expressed in mm

	C		Ob		
LV	10 mm	9 mm	6 mm	8 mm	13 mm
LV partition	2 mm	2 mm	1 mm	1 mm	1 mm
LV wall	2 mm	2 mm	2 mm	2 mm	1 mm
Liver	Normal	Normal	Nonalcoholic fatty liver	Nonalcoholic fatty liver	Nonalcoholic fatty liver
Skeletal muscle system	Normal	Normal	Without changes	Fat infiltration	Cystic lesions
Other	Normal	Normal			Pelvic dilation at the bilateral renal level

**Fig. 3:**
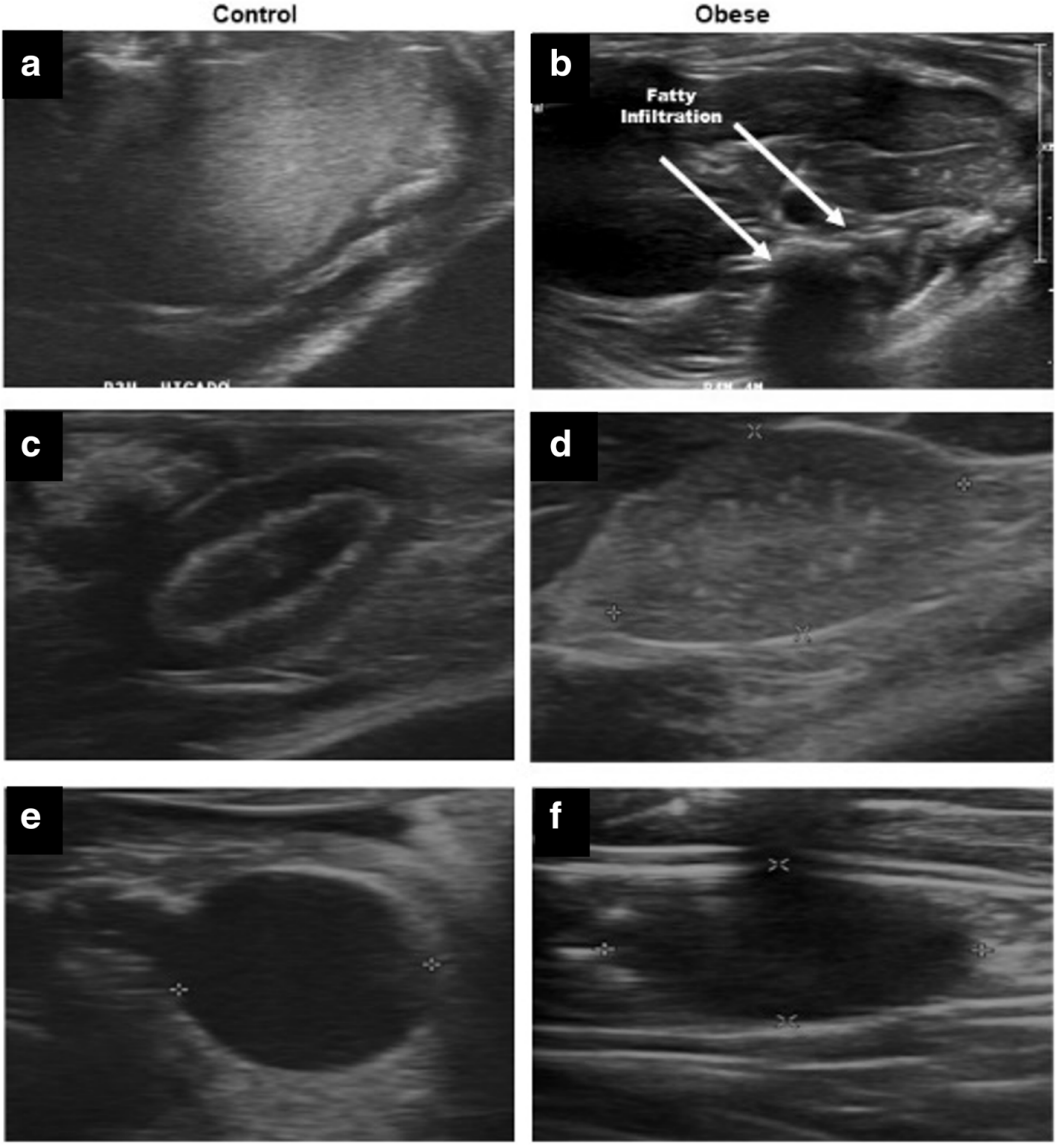
Two-dimensional echocardiography. **a** The right hepatic lobe with fatty infiltration and a homogenous increase in echogenicity in the Ob group. **b** The hepatic parenchyma was homogeneous and isoechoic in the control group. **c** The right kidney in a longitudinal section, where the pelvic dilation can be observed in the Ob group. **d** Another specimen of the right shows an echogenic renal sinus, with no evidence of dilation in the control group. **e** In both images, there is a level approach to the distal joints of the hind legs of the rodents where cystic lesions were found. Two-dimensional echocardiography: In both images, the approach is at the level of the distal joints of the hind legs of the rodents; cystic lesions were found to be predominant in the Ob group. **f** Damage from these lesions can be observed

However, in Fig. 3, the analysis of the two-dimensional ultrasound was more complete, revealing fat infiltration with the presence of a fatty liver (Table 3; Fig. 3b) in the obese group; on the other hand, an isoechoic homogeneous hepatic parenchyma was found in the control group (Fig. 3a).

In another analysis of the ultrasound dimensions, the Ob group showed renal pelvic dilatation compared to the control group, in which the kidney was echogenic, with no evidence of dilation (Fig. 3c, d).

In the Ob group, the ultrasound images revealed musculoskeletal alterations that were due to fat deposits and demonstrated the presence of articular cysts, in the posterior region of the distal joints, where it was possible to view the damage caused the accumulated fat (Table 3; Fig. 3e, f).

## Discussion

In this study, we show the relationships among obesity, endothelial dysfunction and alterations in several organs, such as the musculoskeletal system, kidney, liver and heart, through noninvasive methods such as echography. Thus, our results revealed that a high fat-fructose diet increased food intake, with marked weight gain due to the caloric contribution provided by fat and carbohydrates in obese group. We also found metabolic alterations, such as increases in glucose, triglycerides, cholesterol and the HOMA-IR index, similar to other studies that have used hypercaloric diets [13, 15]. Hence, our results showed that with obesity triggered dysmetabolism, hyperglycemia and hypertriglyceridemia, as well as an increase of the levels of inflammatory cytokines (TNF-α, IL-6), are present. Likewise, as a consequence of obesity, alterations in other organs can manifest and these changes can be analyzed by ultrasound. In this work the results at the muscle-skeletal and articular levels showed the presence of articular cysts, in the posterior region of the distal joints, where it was possible to view the damage due to fat accumulation. Other alterations were present in the heart, kidney, musculoskeletal system and liver analyzed by echography.

In this study, we used a noninvasive and rapid method, to evaluate the ventricular anatomy. Although the cut-off point for normal relative parietal thickness in humans is 0.42 mm, this criterion was highly specific in our animals because the rats presented a relative parietal thickness below that limit [11, 12]. Additionally, in this work the presence of Baker’s cyst (QB), or popliteal cyst, was first described in 1840 by Adams [16] and later by Baker [17]. In 1877, Baker published his experience with this entity, which caused this type of cyst to be named after him. Baker’s cyst is defined as an abnormal cluster of synovial fluid in the gastrocnemius-semimembranosus bursa or as a herniation of the posterior joint capsule with synovial fluid tension [17–19]. Changes in both static and dynamic alignment of the lower extremities could alter balance and gait, and trigger pain throughout the lower limbs [20]. In addition, ultrasound changes in the liver indicated fatty liver in 75% (6 exemplary) of the obese rats. One of the main organs affected by obesity is the liver, where long-term increases in lipogenesis and decreases in mitochondrial β-oxidation of nonesterified fatty acids, as well as hepatic triglyceride secretion, can contribute to fat accumulation in the liver, leading to the appearance of liver steatosis. Additionally, in a prospective longitudinal study, 86% of patients with NAFLD and progressive fibrosis were obese [14, 15, 21].

At the renal level, changes such as pelvic dilation were found in 50% of the obese group rats. On the other hand, changes were observed at the level of the musculoskeletal system with the presence of joint cysts in the posterior region of the distal lower-extremity joints of the rodents. Thus, excessive body weight creates greater load stress, which causing joint misalignment (deformities) in the lower extremities and inflammatory and degenerative processes. This condition could decrease physical functioning due to associations with mobility and pain [22].

Several studies have shown that the mechanical stress caused by overload or repetitive use can trigger tendon pathology. Moreover, certain extrinsic factors (posture and activity) and intrinsic factors (genetics and metabolic characteristics) can interfere with their development [16]. This condition and metabolic factors (hyperglycemia, dyslipidemia and endothelial dysfunction) affect the quality of life of subjects.

Regarding the inflammatory process and endothelial dysfunction, we also found an increase in proinflammatory cytokines, such as TNF-α and IL-6, which augment monocytes adhesion to endothelial cells. It is known that in both; humans and mice, an imbalance between TNF-α and adiponectin (an adipokine of adipose tissue) participates in the progression to steatohepatitis [23–25]. Therefore, adipose tissue releases inflammatory mediators, proinflammatory molecules such as TNF-α and IL-6, and some other mediators such leptin, adiponectin, and resistin. IL-6, TNF-α and leptin act on immune cells and cause local and systemic inflammation. In several studies, endothelial damage has been demonstrated to augment molecules such as monocyte attractant chemoprotein 1 (MCP-1), ICAM-1, VCAM-1 and plasminogen activator inhibitor (PAI-1), which contribute to vascular complications [6, 7, 26, 27].

Thus, our results of adhesion molecules showed an increase in the Ob group; this confirms endothelial damage, which has an impact on the joints. In addition, ICAM-1 and VCAM-1 are known to be activating molecules of endothelial dysfunction because they play a crucial role in the adhesion of cells to the endothelial surfaces and in the integrity of the vascular wall, generating an accumulation of cells and sparking oxidative stress, which can be modulated by the body composition and eating pattern [4, 6, 28, 29].

## Conclusion

In this study, we observed a relationship between endothelial dysfunction and the changes observed at the level of the musculoskeletal system, liver and heart with the presence of articular cysts in the posterior region of the distal lower-extremity joint in obese rodents. Thus, we suggest that ultrasound is an excellent diagnostic tool that is accessible and easy to use to access the chronic diseases that are increasing in our population. Moreover, ultrasound allows an integral assessment of the alterations in different organs, joints and tissues, as well as, better monitoring and support to reduce complications and improve the quality of life of patients.

## Supplementary information

**Additional file 1.**

## Data Availability

The datasets used and/or analyzed during the current study are available the supplementary file Results Rats BMC Endocrine.

## Abbreviations

AO: Aorta
AE: Left atrium
CAMs: Cellular adhesion molecules
C: Control group
ELISA: Enzyme immunoassay
HDL: High Density Lipoprotein
HOMA-IR: Homeostasis Model Assessment-Insulin Resistance Index
ICAM-1: Intercellular Adhesion Molecule-1
IL-6: Interleukine-6
IMSS: Mexican Social Security Institute
LV: Left ventricle
LDL: Low Density Lipoprotein
MCP-1: Monocyte Attractant Chemoprotein 1
NAFLD: Non-Alcoholic Fatty Liver Disease
Ob: Hypercaloric diet group
PAI-1: Plasminogen Activator Inhibitor
QB: Baker’s cyst
RV: Right ventricle
SD: Standard deviation
TG: Triglycerides
TNF-α: Tumor Necrosis Factor-α
VCAM-1: Vascular Adhesion Molecule-1
VLDL: Very Low Density Lipoprotein
